# The efficacy of a transdiagnostic sleep intervention for outpatients with sleep problems and depression, bipolar disorder, or attention deficit disorder: study protocol for a randomized controlled trial

**DOI:** 10.1186/s13063-024-07903-6

**Published:** 2024-01-16

**Authors:** Mette Kragh, Henny Dyrberg, Maria Speed, Pernille Pedersen, Sanne Toft Kristiansen, Klaus Martiny

**Affiliations:** 1 Department of Affective Disorders, Aarhus University Hospital Psychiatry, Evald Krogs Gade 13A, 8000 Aarhus C, Denmark; 2 DEFACTUM, Central Denmark Region, Evald Krogs Gade 16A, 8000 Aarhus C, Denmark; 3 Research Unit for Nursing and Healthcare, Department of Public Health, Aarhus University, Bartholins Allé 2, 8000 Aarhus C, Denmark; 4 Mental Health Centre Copenhagen, University Hospital Copenhagen, Hovedvejen 17, 2000 Frederiksberg, Denmark; 5 Department of Clinical Medicine, University of Copenhagen, Copenhagen, Denmark

**Keywords:** Insomnia, Circadian rhythm disorder, Hypersomnia, Depression, Bipolar affective disorder, Attention deficit disorder, Actigraphy, Randomized controlled trial

## Abstract

**Background:**

Patients with mental disorders have a higher prevalence of sleep problems than the general population. Sleep problems may include insomnia, circadian rhythm disorders, or hypersomnia. A transdiagnostic approach combining cognitive behavioral therapy for insomnia (CBT-I) with chronotherapy addressing a broad range of sleep problems has shown promising results in a limited number of studies. The aim of the study is to investigate the efficacy of a transdiagnostic sleep intervention for patients with sleep problems comorbid to bipolar disorder, unipolar depression, or attention deficit disorders. The primary hypothesis is that the intervention improves sleep quality compared with a control group. The secondary hypotheses are that the intervention increases subjective and objective sleep efficiency, reduces sleep onset latency, wake after sleep onset, number of awakenings, and severity of insomnia; and that it improves well-being, personal recovery, work ability, and consumption of sleep medication compared with a control group.

**Methods:**

The study is a randomized controlled trial enrolling 88 outpatients with bipolar disorder, major depression, or attention deficit disorder with symptoms of various sleep problems (insomnia, circadian rhythm disorders, or hypersomnia). Patients are allocated to either an intervention group receiving six sessions of transdiagnostic sleep treatment or to a control group receiving a single session of sleep hygiene education. Assessments are made at baseline, at week two, and after 6 weeks in both groups. Actigraphy is performed continuously throughout the 6-week study period for all patients. The primary outcome is changes in the subjective appraisal of sleep quality (Pittsburgh Sleep Quality Index). The secondary outcomes are changes in sleep efficiency, sleep onset latency, wake after sleep onset, number of nocturnal awakenings (based on actigraph and sleep diary data), changes in insomnia severity (Insomnia Severity Index), well-being (WHO-5 Well-Being Index), personal recovery (INSPIRE-O), work ability (Work Ability Index), and consumption of sleep medication (sleep-diaries).

**Discussion:**

The study was initiated in 2022 and the inclusion period will continue until mid-2024. The results may have implications for the development and implementation of additional treatment options for patients with mental disorders and comorbid sleep problems.

**Trial registration:**

ClinicalTrials.gov. NCT05406414. Registered on June 6, 2022.

**Supplementary Information:**

The online version contains supplementary material available at 10.1186/s13063-024-07903-6.

## Administrative information

Note: the numbers in curly brackets in this protocol refer to SPIRIT checklist item numbers. The order of the items has been modified to group similar items (see http://www.equator-network.org/reporting-guidelines/spirit-2013-statement-defining-standard-protocol-items-for-clinical-trials/).

**Table:** 

Title {1}	The efficacy of a transdiagnostic sleep intervention for outpatients with sleep problems and depression, bipolar disorder, or attention deficit disorder: study protocol for a randomized controlled trial
Trial registration {2a and 2b}.	The study was approved by the ethical committee in the Central Denmark Region, record number 1–10-72–38-22. The project followed the General Data Protection Regulation (EU) and is registered with the Internal Record of Research Projects at the Central Denmark Region, record number: 1–16-02–151-22. Furthermore, the study is registered with ClinicalTrials.gov, Identifier: NCT05406414 as from June 6, 2022.
Protocol version {3}	Protocol Version 02, March 31, 2022.
Funding {4}	This study is supported by the Public Health in Central Denmark Region Foundation, the Jascha Foundation, the Legacy of Director Werner Richter and Spouse Foundation and by the Department of Affective Disorders, Aarhus University Hospital Psychiatry, Denmark, and the Municipality of Aarhus, Denmark.
Author details {5a}	Mette Kragh*^1^, Henny Dyrberg^1^, Maria Speed^1^ Pernille Pedersen^2^, Sanne Toft Kristiansen^3^, Klaus Martiny^4^ ^1^Department of Affective Disorders, Evald Krogs Gade 13A, 8000 Aarhus C, Aarhus University Hospital Psychiatry, Denmark: Mette Kragh (corresponding author), mekrag@rm.dk *; Henny Dyrberg, henndyrb@rm.dk; Maria Speed, MARSPD@rm.dk. ^2^ DEFACTUM, the Central Denmark Region, Evald Krogs Gade 16A, 8000 Aarhus C, Denmark, Pelped@rm.dk. ^3^ Research Unit for Nursing and Healthcare, Department of Public Health, Health, Aarhus University, Bartholins Allé 2, 8000 Aarhus C, Denmark, stk@clin.au.dk. ^4^ Mental Health Centre Copenhagen, University Hospital Copenhagen Hovedvejen 17, 2000 Frederiksberg, Denmark, and Department of Clinical Medicine, University of Copenhagen, Denmark, Klaus.Martiny@regionh.dk.
Name and contact information for the trial sponsor {5b}	Head Consultant Farahna Harees, Department of Affective Disorders, Palle Juul-Jensens Boulevard 175, DK-8200 Aarhus N, Denmark.
Role of sponsor {5c}	The sponsor has approved the study design, but has no authority regarding the collection, management, analysis, or interpretation of the data; the writing of the report; or the decision to submit the report for publication. The same applies to all funding bodies.

## Introduction

### Background and rationale {6a}

The estimated prevalence of chronic insomnia falls in the 10–20% range in the general population [1], and chronic insomnia is associated with increased risk of cardiovascular disease [2], diabetes [3], impaired quality of life [4], increased suicidal ideation [5], increased risk of depression [6], and impaired work functioning [7]. Insomnia often presents as comorbidity to a mental disorder. Regardless of psychiatric diagnoses, insomnia is found in nearly 50% of patients [8], and the incidence of low sleep quality is even higher, close to 90%, for patients with depression [9]. Apart from insomnia, circadian rhythm disorders, hypersomnia, and insufficient sleep hygiene practices may affect sleep [10].

Circadian rhythm disorders with a preference for later bedtime are common in patients with unipolar depression (UD) [11], bipolar disorder (BD) [12], and attention deficit disorders (ADHD) [13]. Patients tend to go to bed later than 2am and sleep during the daytime, resulting in a delayed or irregular sleep phase causing social jetlag [10]. Misalignment with the light/dark cycle and hypersomnia are common problems in patients with BD, affecting approximately 25% of individuals between episodes BD [14]. Patients with UD may spend too much time in bed using sleep as an escape from troublesome symptoms such as anhedonia, lack of energy, and motivation. Similarly, patients with ADHD often find it hard to get out of bed because of initiation problems [15]. This may result in daytime inactivity and poor sleep hygiene [16]. Furthermore, poor sleep hygiene, e.g., exposure to blue light in the evening, is common in youth across mental disorders [10]. Apparently, real-life sleep problems are interwoven and complex [17]. Treatment for circadian rhythm disorders, hypersomnia, and insufficient sleep hygiene, as well as insomnia, is essential. Furthermore, interventions should be personalized to accommodate each patient´s unique sleep pattern [10].

Sleep problems are believed to play an important role in the onset, course, and treatment of UD, BD, and ADHD, with evidence suggesting a bidirectional relationship [6, 18–20]. Pharmacological sleep treatment is widespread but recommended for short-term use only due to the inherent risk of developing tolerance and addiction [21]. European and American guidelines recommend non-pharmacological treatment such as cognitive behavioral therapy for insomnia (CBT-I) as a first-choice treatment for chronic insomnia [21, 22]. CBT-I involves various interventions, including sleep restriction (limit time in bed to the actual sleep time); stimulus control method (strengthen the connection between bed and sleep); sleep hygiene education; relaxation training (reduce physiological arousal), and cognitive methods to address dysfunctional beliefs about sleep. CBT-I can be delivered in various treatment formats, including group, individual [23], and digital formats [24]. Meta-analyses found that CBT-I targeting insomnia with comorbid disorders had a medium to large effect on subjective sleep variables [25, 26].

The present intervention study is inspired by a transdiagnostic manual combining CBT-I with chronotherapy (treatment methods used to influence the circadian rhythm, such as bright light therapy and social rhythm therapy) in conjunction with motivational interviewing [27]. The manual provides transdiagnostic sleep interventions across a wide range of mental disorders [10]. Few studies have examined the effect of transdiagnostic sleep interventions. A positive effect on sleep parameters was shown in adolescents with various mental illnesses [28] and in 14 adolescents with ADHD [29]. Studies in adult outpatients with mental disorders and also midlife and older adults with serious mental illness documented a positive effect on sleep and circadian rhythm parameters and an improvement of psychiatric symptoms [30, 31]. However, despite extensive research on CBT-I, there is limited evidence of the effectiveness of combining CBT-I with chronotherapy as a transdiagnostic intervention in adult patients suffering from UD, BD, and ADHD. Therefore, more evidence is needed to adapt and incorporate the most effective interventions for sleep problems in mental healthcare services.

### Objectives {7}

The aim of the study is to investigate the efficacy of a transdiagnostic sleep intervention for patients with sleep problems comorbid to bipolar disorder, unipolar depression, or attention deficit disorders.

The primary hypothesis is that the transdiagnostic sleep intervention improves sleep quality compared with a control group receiving brief sleep hygiene education.

The secondary hypotheses are that a transdiagnostic sleep intervention increases subjective and objective sleep efficiency (SE), reduces sleep onset latency (SOL), wake after sleep onset (WASO), and nocturnal awakenings (NWAKE); and that it reduces the severity of insomnia, while increasing well-being, personal recovery, and work ability. Furthermore, participation in the intervention is expected to result in a reduction in the consumption of sleep medication compared with a control group receiving short sleep hygiene education.

### Trial design {8}

The study is a randomized controlled trial enrolling 88 outpatients with sleep problems (symptoms of chronic insomnia, circadian rhythm disorders, or hypersomnia) comorbid to BD, UD or ADHD. Patients are allocated to two parallel groups; an intervention group receiving six sessions of transdiagnostic sleep treatment or a control group receiving one session of sleep hygiene education. The allocation ratio is 1:1. After 6 weeks, the control group is offered the sleep intervention, but the data collected from this group will not be included in the present project.

## Methods: participants, interventions, and outcomes

### Study setting {9}

Patients are recruited from the outpatient unit entitled “Psykiatriens Hus,” organizationally located under the Department of Affective Disorders, Aarhus University Hospital Psychiatry, Central Denmark Region, Denmark. The patients referred to treatment for sleep problems are screened for the inclusion and exclusion criteria in connection with their first consultation. The referred patients receive treatment for their mental disorder at different out-patient’s clinics. Patients are generally referred to sleep treatment at “Psykiatriens Hus,” when they are relatively well treated and their mental disorder is stable.

### Eligibility criteria {10}

#### Inclusion criteria

We include patients with:

- A diagnosis of F31 bipolar disorder, F32-33 unipolar depression, or F90 attention deficit disorder (ADHD, ADD) according to The International Classification of Diseases, tenth version (ICD-10).
- Age ≥ 18 years
- Insomnia Severity Index (ISI) ≥ 14 (corresponding to the sleep problem/insomnia threshold)

Sleep problems for 3 months: at least one of the following three times a week or more:

- SOL ≥ 30 min
- WASO ≥ 30 min
- Total sleep time ≥ 11 h per day
- Displaced circadian rhythm; sleeps earlier than 8 pm or later than 02am
- Irregular circadian rhythm: bedtime varies ≥ 3 h throughout the week.

#### Exclusion criteria

- Acutely increased suicide risk (the Central Denmark Regions guideline on assessment of suicide risk)
- Active substance abuse (F10–19)
- The sleep problem in the individual can be attributed to inadequate treatment of physical diseases affecting sleep, e.g., chronic pain condition (documented in the patients’ electronic journal)
- Unstable social situation (does not have a permanent residence)
- Shift work (≥ 2 times a week for the past 2 months)
- Pregnancy and breast-feeding
- Participation in other psychiatric research interventions during the intervention period.

Symptoms of chronic insomnia disorder is defined as difficulties initiating or maintaining sleep or early morning awakenings for at least three months [32]. Standard criteria are defined as SOL or WASO for more than 30 min three times a week or more [33]. Symptoms of circadian rhythm sleep–wake disorders include delayed sleep phase disorder characterized by a sleep pattern delayed by more than 2 h compared to the socially acceptable bedtime and circadian sleep–wake disorder not otherwise specified, e.g., an irregular sleep–wake pattern [32]. For practical reasons, we define displaced circadian rhythm as sleeps earlier than 8 P.M. or later than 02 A.M. and the irregular sleep–wake pattern as a bedtime variability > 3 h. Both definitions are inspired by a study protocol by Harvey et al. [17]. Patients suffering from symptoms of hypersomnia associated with a psychiatric disorder experience excessive nocturnal sleep, daytime sleepiness, poor sleep quality not better explained by other disorders, or medication for more than 3 months [32]. No specific sleep duration is indicated for hypersomnia. In a study protocol by Harvey et al., a sleep length of 9 h was applied [17]. However, in young patients, a sleep length of 9 h is not unusual and we have therefore chosen a duration of 11 h to describe hypersomnia. The included patients are expected to fulfill the criteria of at least one of the following sleep disorders according to ICD-10; F51.05 Insomnia due to other mental disorder, F51.0 Nonorganic insomnia, F51.1 Nonorganic hypersomnia, F51.2 Nonorganic disorder of the sleep–wake schedule.

### Who will take informed consent? {26a}

Informed consent will be obtained from all patients before participation in the study. Patients are recruited through their primary contact nurse, doctor, or psychologist, who provide brief oral and written information about the research project. Furthermore, the primary contact person will obtain the patient's approval for establishing contact between the patient and a project member for a meeting where more thorough information will be given; patients are informed about their right to bring a companion or relative to this meeting. After the meeting, patients will be offered a 1–2-day reflection period before deciding whether to participate in the project or not. Hereafter, informed oral and written consent are obtained. Participation is voluntary, and patients may choose to withdraw from the project at any time. The patients will not receive any payment for participating in the study.

### Additional consent provisions for collection and use of participant data and biological specimens {26b}

Not applicable as no additional content is needed in this study.

### Interventions

#### Explanation for the choice of comparators {6b}

Patients in the control group receive individual sleep hygiene education consisting of a single session presenting the “10 Tips for Better Sleep” (see Additional file 1). They receive a flyer containing advice on how to promote good sleep practices and must work independently with these tips for the next 6 weeks while waiting for transdiagnostic sleep treatment. As a stand-alone intervention, sleep hygiene education has shown no effect on insomnia [34] and it is therefore chosen as the intervention given in the control group. Thus the intervention can be considered a sham treatment. However, sleep hygiene education is also often treatment as usual for this patient group.

#### Intervention description {11a}

The intervention group receives transdiagnostic sleep treatment consisting of six individual sessions lasting 60 min each in the course of 6 weeks. The sessions are conducted by a team of nurses, a physiotherapist, an occupational therapist, and a psychologist. These professionals have undergone a minimum of a two-day intensive theoretic sleep course to enhance their knowledge and skills in the field of sleep. Subsequently, they have received peer-to-peer training. Training and supervision are provided by the first and second authors of this paper.

The sessions have the following contents:

1. Assessment and introduction to the sleep diary. The items from the sleep diary are similar to those of the consensus sleep diary [35]
2. Sleep restriction, sleep compression, stimulus-control, or phase advance. Setting goals [10]
3. Education about normal sleep, sleep problems, and circadian rhythm
4. Cognitive techniques (e.g., behavioral experiments) [10]
5. Introduction to relaxation training (e.g., progressive muscle relaxation) [36]
6. Follow-up on goals and plan for preventing sleep problem relapse

### Criteria for discontinuing or modifying allocated interventions {11b}

The allocated intervention will be discontinued on the patient’s request, and patients may withdraw from the study for any reason. The investigators have the discretion to withdraw patients from the study if their mental disorder is deteriorating, e.g., if they experience a manic episode, are at an acutely increased suicide risk, or if they are hospitalized for any reason.

### Strategies to improve adherence to interventions {11c}

Protocol adherence is handled by the first and second author with assistance from a research assistant who secures that all required questionnaires and all items of information are entered into the Research Electronic Data Capture (REDCap) 8.5.22 2019 Vanderbilt University system.

### Relevant concomitant care permitted or prohibited during the trial {11d}

This study is an add on to relevant concomitant treatment, which is allowed during the study period. Concomitant treatment includes sessions with the primary contact nurse to evaluate the effect of pharmacotherapy. Participation in other psychiatric research interventions during the intervention period is prohibited. Treatment with hypnotics are allowed during the study and will be registered. The hypnotics-use in both groups will be compared statistically to test for significant differences.

### Provisions for post-trial care {30}

Any harm to patients from attending the trial is covered by the Danish patient reimbursement scheme. After the trial has concluded, patients will continue their usual treatment.

### Outcomes {12}

#### Primary outcome

Changes in the quality of sleep measured by the Pittsburgh Sleep Quality Index (PSQI) [37].

Data are obtained by questionnaires at inclusion (baseline/week 0), at week 2, and at week 6.

#### Secondary outcomes

1. Changes in SE are measured daily during the 6-week study period. SE changes are calculated by measurements from actigraphy and registrations in sleep diaries. More specifically, SE is calculated as; total sleep time (TST)/time in bed (TIB) × 100. In the actigraphy data, TST is calculated by the Motionware software, and TIB is defined as the period from the patient presses the event marker at “lights out” until they get out of bed (time registered in the sleep diary). The calculation of SE based on sleep diary data is based on the TST and TIB registered by the patient in the sleep diary.
2. SOL changes. SOL is the length of time it takes to accomplish the transition from full wakefulness to sleep, and it is measured daily during the 6-week study period. SOL is calculated based on the patient's registered bedtime (“lights out”) marked by the event marker and the first sleep onset measured by actigraphy. In sleep diaries, SOL is calculated based on the patient's registered bedtime (“lights off”) and their registered fell-asleep-time.
3. Changes in WASO are measured daily during the 6-week study period. WASO changes are calculated by measurements from actigraphy as the difference between assumed sleep and actual sleep time. Furthermore, WASO is calculated based on patients’ registrations in sleep diaries, where they report how long their nocturnal awakenings last.
4. Changes in the number of NWAKE are measured daily during the 6-week study period. NWAKE is calculated by measurements from actigraphy (called “wake bouts”) and based on the sleep diaries where patients register the number of NWAKE. The night-time sleep frame in actigraphy data is defined as sleep occurring during a 16-h night interval (from 20:00 h to 11:59 h). The investigators designate the first sleep episode after 8PM as the first nocturnal sleep episode. If the individual is already asleep at that time, the bedtime will be moved earlier to the first wake epoch preceding sleep onset. If the individual is still asleep after 11:59PM, wake time will be moved to the first subsequent epoch of activity.
5. Changes in the subjective appraisal of insomnia severity. Data are measured by questionnaires at inclusion (baseline/week 0), at week 2, and at week 6 (endpoint). The insomnia severity status is measured by the Insomnia Severity Index (ISI) [38].
6. Changes in the subjective experience of well-being. Well-being is measured at inclusion (baseline/week 0), at week 2, and at week 6 (endpoint) by the WHO-5 Well-Being Index (WHO-5) questionnaire [39].
7. Changes in level of personal recovery. These changes are measured at inclusion (baseline/week 0), at week 2, and at week 6 (endpoint) by the INSPIRE-O questionnaire [40].
8. Changes in work ability are measured at inclusion (baseline/week 0), at week 2, and at week 6 (endpoint) by the Work Ability Index [41] questionnaire.
9. Changes in the consumption of sleep medication are registered daily during the 6-week study period. Daily use of sedatives and hypnotics will be measured by registration of medication use in sleep diaries and registrations in the electronic health record made during the study period [time frame: 6 weeks = 42 days]. The investigators will measure the change in total use of regular and per-need sedatives and hypnotics in mg.

### Participant timeline {13}

**Fig. 1:**
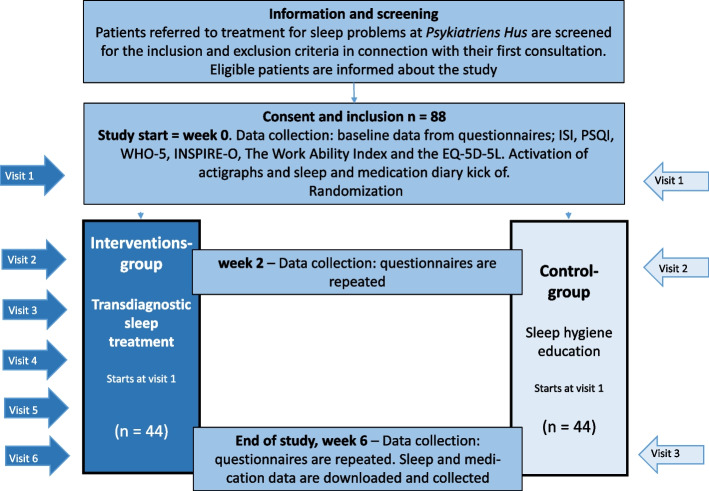
Participant timeline. Abbreviations: Insomnia Severity Index (ISI), Pittsburgh Sleep Quality Index (PSQI), WHO-5 Well-Being Index (WHO-5)

The participant timeline is shown in Fig. 1.

### Sample size {14}

A power calculation was performed based on the results of a similar study [28] with PSQI as a secondary outcome using an average effect size of 0.67. Assuming a power of 0.80, a significance level of 0.05, and an expected power magnitude of 0.67, a sample size of *n* = 44 is obtained for each group. The expected 25% dropout rate is based on former experiences from a pilot project (*n* = 55). A total of 88 patients will be included in the study.

### Recruitment {15}

Patients are recruited among patients referred with sleep problems to “Psykiatriens Hus.” Patients may be referred to this intervention for sleep problems from several mental health outpatient clinics at Aarhus University Hospital, Psychiatry, and from two Municipal counseling services in Aarhus. Based on experiences from a previous pilot project, we expect 100 annual referrals of which approximately 50% will likely be included in the study. If the inclusion rate is unexpectedly low, the research team will visit the referring staff and advertise to boost participation in the project. If necessary, the expected 1.5–2-year duration of the enrolment period will be extended.

### Assignment of interventions: allocation

#### Sequence generation {16a}

All included patients will attend baseline assessments prior to randomization into the two study groups using block randomization with varying block sizes of 4, 6, and 8. Randomization will be set up in REDCap by an independent service provider.

#### Concealment mechanism {16b}

An investigator will be informed of allocation when pressing the randomization button in REDCap. However, detailed information on block size is not available.

#### Implementation {16c}

The investigator will inform the patients about their allocation and introduce them to either the intervention or the control group.

### Assignment of interventions: blinding

#### Who will be blinded {17a}

Blinding of allocation is not possible because of the nature of the intervention.

#### Procedure for unblinding if needed {17b}

Not applicable. Blinding is not possible because of the nature of the intervention. The data analysis will be performed blinded to the allocated intervention group.

### Data collection and management

#### Plans for assessment and collection of outcomes {18a}

Patients who discontinue study participation + / − 7 days from the planned follow-up at 2 or 6 weeks will be asked whether they wish to complete the questionnaires when they return the actigraph. The PSQI and ISI questionnaires will be prioritized.

#### Plans to promote participant retention and complete follow-up {18b}

Patients who deviate from intervention protocols due to, e.g., illness, are asked to complete the questionnaires as soon as possible. We offer to do this with the patients by phone, at a home visit or virtually based on the patient’s preference. We also allow a flexibility of + / − of 7 days from the planned follow-up visits.

#### Data management {19}

The following data will be collected:

1. From the patients and their hospital records: name, phone number, personal identification number from the Danish Civil Registration System (“CPR number”), diagnosis, and medication prescriptions. The following additional data are included through a semi-structured assessment interview at inclusion: marital status, employment status, level of education, consumption of tobacco, alcohol, other drugs, previous treatment for sleep problems, and exposure to daylight are collected.
2. From actigraphy (Actiwatch MotionWatch 8 from CamNtech Ltd, Cambrigde UK): SE, SOL, WASO, and NWAKE. The MotionWatch 8 is a wrist-worn activity monitoring device, similar in size to a watch, with a tri-axial accelerometer. It has a 4.0 Mbits of storage capacity and a waterproof casing. It is used to objectively monitor movement during sleep. For sleep scoring, a tri-axial accelerometer is used to monitor body movements. For differentiating between sleep and wake epochs, the MotionWare Software (version 1.2.28, CamNtech, Ltd.) is used. This device uses an algorithm that depends on thresholding, assigning an activity score to each epoch by totalizing the epoch in question and those surrounding it using weighting factors. If the activity score of an epoch is above a predefined threshold, the epoch is scored as wake; otherwise, it is scored as sleep. A 30-s epoch is used in this study. Patients are asked to wear the actigraph 24 h a day on their non-dominant wrist during the 6-week study. Patients are instructed to press the event-marker when they intend to sleep (“lights out”) and when they experience their final wake up in the morning. Sleep diaries are used to validate sleep and wake onset if the patients forget to press the eventmarker. The first author and an experienced sleep scientist will perform the scoring and analyze the actigraphic records. The degree of agreement between raters in ten scorings will be calculated and reported using the intraclass correlation coefficient.
3. From sleep diaries: The use of sleep medication “as needed”, bedtime (“lights out”), sleep onset, wakeup time and time for getting out of bed, SE, SOL, WASO, NWAKE, daytime naps including lengths (Additional files 2 and 3: Sleep and medication diary for the intervention and control group).
4. From six validated questionnaires:
  The ISI measures the subjective experience of insomnia severity. The scale ranges from 0 to 28. The higher the score, the more severe the insomnia [38].
  The PSQI measures sleep quality [37]. The score ranges from 0 to 21, “0” indicating no difficulty and “21” indicating severe difficulties.
  The WHO-5 describes well-being (38). The questionnaire measures well-being on a scale ranging from 0 to 100. The higher the score, the more well-being.
  The INSPIRE-O consists of five short questions that measure personal recovery [40]. The questionnaire measures personal recovery on a scale ranging from 5 to 25. The higher the score, the more recovery.
  The Work Ability Index, a questionnaire measuring work ability [41]. A score of 0 shows the lowest possibility that the person would take on a job; a score of 10 denotes the highest probability.
  The International EQ-5D-5L questionnaire estimates quality-adjusted years of life. The questionnaire is applied to estimate quality-adjusted years of life [42].

All patients complete the six validated questionnaires at personal visits at baseline (week 0) and again after 2 and 6 weeks. Patients are asked to wear actigraphs around the clock during all 6 weeks and to complete a short sleep diary on a daily basis (see Additional files 2 and 3). Furthermore, they complete the morningness-eveningness questionnaire (MEQ) at week 2 to estimate their chronotype. This validated questionnaire has scores ranging from 16 to 86. Scores of 41 and below indicate “evening types.” Scores of 59 and above indicate “morning types.” Scores between 42 and 58 indicate “intermediate types” [43]. For the study schedule used for enrolment, intervention, and assessments, please see Table 1.

**Table 1:**
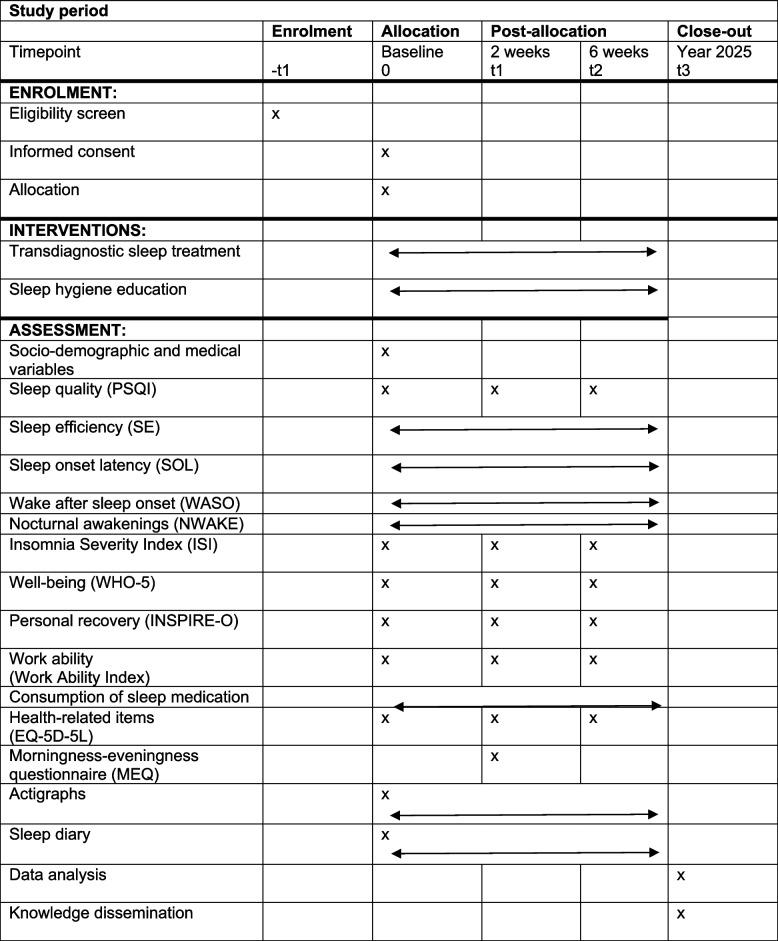
Study schedule used to record enrolment, intervention, and assessment

All data will be stored in REDCap; a secure browser-based, metadata-driven EDC software solution and workflow methodology to design clinical and research databases [44].

### Confidentiality {27}

Data will be entered and securely stored in REDCap. Data will be fully anonymized before being shared with co-authors and the statistician at the Department of Affective Disorders at Aarhus University Hospital, Denmark. The trial will be conducted in conformity with the Declaration of Helsinki—Ethical principles for medical research involving human subjects.

### Plans for collection, laboratory evaluation, and storage of biological specimens for genetic or molecular analysis in this trial/future use {33}

Not applicable, no samples will be collected.

### Statistical methods

#### Statistical methods for primary and secondary outcomes {20a}

Demographic and clinical data used to compare the intervention and control group will be analyzed using descriptive statistics. Categorical variables will be reported using proportions, whereas means and standard deviations will be reported for continuous variables. Fisher’s exact test and Student’s *t*-test are applied to test for the difference between the intervention and control group for categorical and continuous variables. The intent-to-treat method will be used to examine if a significant change in the ISI and PSQI total scores from baseline to 6 weeks can be established.

To compare the differences in measurements between the intervention and control group over time, we will apply a linear mixed effects (LME) model, which is recommended for longitudinal data with missingness [45]. The “maximum likelihood estimation” assuming “missing at random” for data is used to examine the primary and secondary continuous objectives. The fixed components of the model are gender, age, time variable (1: baseline, 2: 2 weeks, 3: 6 weeks), type of treatment (1: intervention group, 2: control group), and the interaction between the time variable and the type of treatment. Means, standard deviation, 95% confidence interval, power magnitude, and the average reduction over time and *p*-value for reduction in the primary and secondary outcomes will be calculated from the model. All statistical analyses will be performed using Stata version 15., R-Studio, or SPSS version 20.0.

#### Interim analyses {21b}

No interim analysis will be performed because of the short duration of the trial and a minimal intervention risk.

#### Methods for additional analyses (e.g., subgroup analyses) {20b}

See Statistical Methods for primary and secondary outcomes.

#### Methods in analysis to handle protocol non-adherence and any statistical methods to handle missing data {20c}

The intent-to-treat method will be used in conjunction with a LME model, which is recommended for longitudinal data with missingness.

#### Plans to give access to the full protocol, participant-level data and statistical code {31c}

Not applicable, We have no plans in relation to this.

### Oversight and monitoring

#### Composition of the coordinating center and trial steering committee {5d}

No data monitoring committee (DMC) will be established for this trial because the trial is short and limited to a single center.

#### Composition of the data monitoring committee, its role and reporting structure {21a}

No DMC will be established for this trial.

#### Adverse event reporting and harms {22}

Patients in the intervention group will be assessed for adverse events at weeks 2 and 6. All cases of serious adverse events will be reported to the Danish Scientific Ethics Committee.

#### Frequency and plans for auditing trial conduct {23}

No auditing trial is planned because of the minimal risk associated with the intervention.

#### Plans for communicating important protocol amendments to relevant parties (e.g. trial participants, ethical committees) {25}

Any modifications to the protocol which may impact on the conduct of the study, potential benefit of the patient or may affect patient safety, including changes of study objectives, study design, patient population, sample sizes, study procedures, or significant administrative aspects will require a formal amendment to the protocol. Such amendment will be agreed upon by the research and follow group, and approved by the Ethics Committee Central Region Denmark prior to implementation. Administrative changes of the protocol are minor corrections and/or clarifications that have no effect on the way the study is to be conducted. These administrative changes will be agreed upon the research group and will be documented in a memorandum.

#### Dissemination plans {31a}

The results will be submitted for publication in an international peer-reviewed journal. Furthermore, results will be presented at both national and international conferences. The patients will, if they wish, receive an e-mail with an abstract presenting the study results.

## Discussion

The strengths of the study are the use of both subjective and objective data. Furthermore, we investigate patients´ daytime functioning, which is important because sleep problems influence quality of life around the clock. Results from this trial will contribute to knowledge on the effect of the intervention and pave the way for implementing the most effective sleep problem interventions in mental healthcare services. However, the study also has some limitations. The PSQI questionnaire is a retrospective 30-day instrument used for inclusion at the first consultation. However, we also use it for measuring short-term, i.e., 2-week, effects. This results in an interval of only 14 days between assessments at baseline and week 2. Therefore, the answers obtained in week two are biased by baseline answers and comparison may be difficult. Perhaps it would have been appropriate to delay this second data collection, but because of the short 6-week study period, the second data collection would then be placed too late in relation to the last time point.

We have no outcomes related to mental disorders, which could have strengthened the design as the relationship between, e.g., insomnia and depression has been described as bidirectional [46]. Two meta-analyses have concluded that in patients with depression and comorbid insomnia, treatment with CBT-I may lead to an improved depression outcome with a medium-to-large effect size [25, 26]. However, the present study enrolls patients with depressive disorder, bipolar disorder, and attention deficit disorder. Therefore, psychopathology outcomes for each patient group would be needed. For this, we would need larger subgroups. Another option would be to assess general psychiatric symptoms across diagnoses [10], but this would not give answers as to whether diagnostically specific symptoms were reduced. Instead, we have chosen to describe day-time functioning and general well-being with other questionnaires.

Patients in the two groups do not have comparable treatment contacts with the research team. As illustrated in Fig. 1, the intervention group had six visits during the study period and the control group had only three. This may potentially cause improvement due to non-specific treatment factors in the intervention group.

Finally, the short follow-up period of only 6 weeks is a limitation. We will be unable to investigate whether the results can be maintained for a longer period, e.g., 6 months or a year. We have chosen this relatively short 6-week follow-up because the sleep treatment investigated has been standard care in our department for some years. It would be difficult and unethical to recruit patients for the study if the patients in the control group needed to wait 6 months for their treatment. We consider 6 weeks a more acceptable timespan. Lastly, investigating real-life sleep problems in a clinical setting is challenging, and the design must be adapted to what is possible in a psychiatric outpatient clinic/real-world framework.

### Trial status

This manuscript reports protocol version number 2 of March 31, 2022. Recruitment of patients was initiated on May 23, 2022, and is expected to complete in 2024.

### Supplementary Information

**Additional file 1.** 

**Additional file 2.**

**Additional file 3.**

**Additional file 4.**

## Data Availability

The data sets analyzed during the present study will be available from the corresponding author on reasonable request.

## Abbreviations

ADHD: Attention deficit hyperactivity disorder
ADD: Attention deficit disorder
BD: Bipolar disorder
CBT-I: Cognitive behavioral therapy for Insomnia
DMC: Data monitoring committee
ISI: Insomnia severity index
LME: Linear mixed effects
MEQ: Morningness-eveningness questionnaire
NWAKE: Nocturnal awakenings
PSQI: Pittsburgh sleep quality index
REDCap: Research electronic data capture
SE: Sleep efficiency
SOL: Sleep onset latency
TIB: Time in bed
TST: Total sleep time
UD: Unipolar depression
WASO: Wake after sleep onset
WHO-5: WHO-5 Well-Being Index
