# Supplementary material for: The efficacy of a transdiagnostic sleep intervention for outpatients with sleep problems and depression, bipolar disorder, or attention deficit disorder: study protocol for a randomized controlled trial
Source: Trials. 2024 Jan 16;25:57. doi: 10.1186/s13063-024-07903-6 (PMC10790522; doi:10.1186/s13063-024-07903-6)
Supplement: Supplementary file 1 — Additional file 1. [file 13063_2024_7903_MOESM1_ESM.pdf]

10 TIPS FOR GOOD SLEEP

1. Do not fear insomnia
It is not unusual to sleep poorly for a period, you often sleep more than you think.

2. Don't solve today's problems at night
Avoid demanding homework in the last hour before bed and instead relax with a book, music or the like.

3. Only go to bed when you are sleepy
There must be a strong association between bed and sleep. Therefore, only use the bed for sleep (and sex).

4. Avoid blue light from screens before bed
LED screens emit blue light, which inhibits the sleep hormone, Melatonin, which can make it harder for you to fall asleep.

5. Get up at the same time every day. Regularity promotes good sleep habits
The brain has an internal clock of approximately 25 hours, which is reset every day when you get up. Therefore, get up at the same time every day, no matter how little or how much you have slept. This also applies on weekends and holidays.

6. Don't sleep during the day
If you have problems sleeping at night, it is advised that you do not sleep during the day. If the total sleep requirement is e.g., 6 hours, and you sleep 2 hours in the afternoon, there are only 4 hours left for sleep in the night.

7. Do not drink coffee, tea, or cola in the evening
All foods with caffeine have an invigorating and stimulating effect. This also applies to chocolate.

8. Don't go to bed hungry or overfull
Hunger or over satiation act as a stimulant and can prevent sleep.

9. Do not use alcohol as a sleeping pill
You often fall asleep faster when you have alcohol in your blood, but if there is alcohol in your body, the normal sleep pattern is suppressed. This means that sleep is shallower with more awakenings. It impairs the quality of sleep, which causes fatigue the next day.

10. Avoid sleeping pills if possible
Some sleeping pills affect you the next day as well, and many sleeping pills lead to habituation. Sleeping pills work better if you take a tablet occasionally rather than every night.

Sleep Clinic, Region hospital Viborg
