# Supplementary material for: The efficacy of a transdiagnostic sleep intervention for outpatients with sleep problems and depression, bipolar disorder, or attention deficit disorder: study protocol for a randomized controlled trial
Source: Trials. 2024 Jan 16;25:57. doi: 10.1186/s13063-024-07903-6 (PMC10790522; doi:10.1186/s13063-024-07903-6)
Supplement: Supplementary file 2 — Additional file 2. [file 13063_2024_7903_MOESM2_ESM.pdf]

Sleep diary, week __

Date

Monday	Tuesday	Wednesday	Thursday	Friday	Saturday	Sunday

Fill in before you go to sleep

1. Went for a walk? Y/N

Monday	Tuesday	Wednesday	Thursday	Friday	Saturday	Sunday

2. Exposed to daylight ? (hours:minutes)

Monday	Tuesday	Wednesday	Thursday	Friday	Saturday	Sunday

3. Did you eat regularly?

Monday	Tuesday	Wednesday	Thursday	Friday	Saturday	Sunday

4. Relaxed in bed/on couch? (hours:minutes)

Monday	Tuesday	Wednesday	Thursday	Friday	Saturday	Sunday

5. Number of caffeinighted drinks?

Monday	Tuesday	Wednesday	Thursday	Friday	Saturday	Sunday

6. Alcohol (number of drinks)

Monday	Tuesday	Wednesday	Thursday	Friday	Saturday	Sunday

7. Did you take a nap? (time and duration)

Monday	Tuesday	Wednesday	Thursday	Friday	Saturday	Sunday

Fill in next morning

8. What did you do before going to bed?

Monday evening	Tuesday evening	Wednesday evening	Thursday evening	Friday evening	Saturday evening	Sunday evening

9. Sleep medication, name and dosage

Monday evening	Tuesday evening	Wednesday evening	Thursday evening	Friday evening	Saturday evening	Sunday evening

10. Lights out at?

Monday evening	Tuesday evening	Wednesday evening	Thursday evening	Friday evening	Saturday evening	Sunday evening

11. Fell asleep at?

Monday evening	Tuesday evening	Wednesday evening	Thursday evening	Friday evening	Saturday evening	Sunday evening

12. How many times did You wake up? (number of awakenings)

Monday Tuesday night	Tuesday-wednesday night	Wednesday-thursday night	Thursday-friday night	Friday-saturday night	Saturday-sunday night	Sunday-mandag night

13. The awakenings lasted? (Wake after sleep onset)

Monday Tuesday night	Tuesday-wednesday night	Wednesday-thursday night	Thursday-friday night	Friday-saturday night	Saturday-sunday night	Sunday-mandag night

14. This morning i woke up at?

Tuesday	wednesday	Thursday	Friday	Saturday	Sunday	Mandag

15. I got out of bed at?

Tuesday	Wednesday	Thursday	Friday	Saturday	Sunday	Mandag

16. Sleep duration? (Total sleep time)

Monday Tuesday night	Tuesday-wednesday night	Wednesday-thursday night	Thursday-friday night	Friday-saturday night	Saturday-sunday night	Sunday-mandag night

17. How would you assess your sleep? (1 = no rest at all, 2 = no rest , 3 = tolerable, 4 = fairly good, 5 = good)

Monday Tuesday night	Tuesday-Wednesday night	Wednesday-Thursday night	Thursday-Friday night	Friday-Saturday night	Saturday-Sunday night	Sunday-Monday night

18. Sleep efficiency (to be calculated in the next session)

Monday	Tuesday	Wednesday	Thursday	Friday	Saturday	Sunday
