# Supplementary material for: The efficacy of a transdiagnostic sleep intervention for outpatients with sleep problems and depression, bipolar disorder, or attention deficit disorder: study protocol for a randomized controlled trial
Source: Trials. 2024 Jan 16;25:57. doi: 10.1186/s13063-024-07903-6 (PMC10790522; doi:10.1186/s13063-024-07903-6)
Supplement: Supplementary file 3 — Additional file 3. [file 13063_2024_7903_MOESM3_ESM.pdf]

# Sleep diary, week __

**Date**

| | | | | | | |
|---|---|---|---|---|---|---|
| Monday | Tuesday | Wednesday | Thursday | Friday | Saturday | Sunday |

1. Did You take a nap? (When and for how long?)

| | | | | | | |
|---|---|---|---|---|---|---|
| Monday | Tuesday | Wednesday | Thursday | Friday | Saturday | Sunday |

2. I took …. mg to sleep before going to bed (add name, dose)

| | | | | | | |
|---|---|---|---|---|---|---|
| Monday evening | Tuesday evening | Wednesday evening | Thursday evening | Friday evening | Saturday evening | Sunday evening |

3. I turned the lights out at …

| | | | | | | |
|---|---|---|---|---|---|---|
| Monday evening | Tuesday evening | Wednesday evening | Thursday evening | Friday evening | Saturday evening | Sunday evening |

4. Fell a sleep at …

| | | | | | | |
|---|---|---|---|---|---|---|
| Monday evening | Tuesday evening | Wednesday evening | Thursday evening | Friday evening | Saturday evening | Sunday evening |

5. I woke up … times during the night

| | | | | | | |
|---|---|---|---|---|---|---|
| Monday-Tuesday night | Tuesday-Wednesday night | Wednesday-Thursday night | Thursday-Friday night | Friday-Saturday night | Saturday-Sunday night | Sunday-Monday night |

6. I was awake for … minutes

| | | | | | | |
|---|---|---|---|---|---|---|
| Monday-Tuesday night | Tuesday-Wednesday night | Wednesday-Thursday night | Thursday-Friday night | Friday-Saturday night | Saturday-Sunday night | Sunday-Monday night |

7. This morning, I woke up at …

| | | | | | | |
|---|---|---|---|---|---|---|
| Tuesday | Wednesday | Thursday | Friday | Saturday | Sunday | Monday |

8. I got up at …

| | | | | | | |
|---|---|---|---|---|---|---|
| Tuesday | Wednesday | Thursday | Friday | Saturday | Sunday | Monday |
