# Supplementary material for: The efficacy of a transdiagnostic sleep intervention for outpatients with sleep problems and depression, bipolar disorder, or attention deficit disorder: study protocol for a randomized controlled trial
Source: Trials. 2024 Jan 16;25:57. doi: 10.1186/s13063-024-07903-6 (PMC10790522; doi:10.1186/s13063-024-07903-6)
Supplement: Supplementary file 4 — Additional file 4. [file 13063_2024_7903_MOESM4_ESM.doc]

**(S2)**

**Informeret samtykke til deltagelse i et sundhedsvidenskabeligt forskningsprojekt.**

**Forskningsprojektets titel:** Bedre søvn uden medicin - et tværsektorielt kvantitativt og kvalitativt studie for voksne patienter med søvnproblemer og psykiske lidelser

**Erklæring fra forsøgspersonen**:

Jeg har fået skriftlig og mundtlig information og jeg ved nok om formål, metode, fordele og
ulemper til at sige ja til at deltage.

Jeg ved, at det er frivilligt at deltage, og at jeg altid kan trække mit samtykke tilbage uden at
miste mine nuværende eller fremtidige rettigheder til behandling.

Jeg giver samtykke til, at deltage i forskningsprojektet og har fået en kopi af dette samtykkeark
samt en kopi af den skriftlige information om projektet til eget brug.

Forsøgspersonens navn: ________________________________________________________

Dato: _______________ Underskrift: ____________________________________________

Hvis der kommer nye væsentlige helbredsoplysninger frem om dig i forskningsprojektet vil du blive informeret. Vil du **frabede** dig information om nye væsentlige helbredsoplysninger, som kommer frem i forskningsprojektet, bedes du markere her: __________ (sæt x)

Ønsker du at blive informeret om forskningsprojektets resultat samt eventuelle konsekvenser for dig?:

Ja _____ (sæt x) Nej _____ (sæt x)

**Erklæring fra den, der afgiver information:**

Jeg erklærer, at forsøgspersonen har modtaget mundtlig og skriftlig information om forsøget.

Efter min overbevisning er der givet tilstrækkelig information til, at der kan træffes beslutning om deltagelse i forsøget.

Navnet på den, der afgiver information:

Dato: _______________ Underskrift: ____________________________________________

Projektidentifikation: ( Fx komiteens Projekt-ID, EudraCT nr., versions nr./dato eller lign.)

version 01 28.01.2022
